# Nitrate Metabolism and Ischemic Cerebrovascular Disease: A Narrative Review

**DOI:** 10.3389/fneur.2022.735181

**Published:** 2022-03-03

**Authors:** Yicong Wang, Weiqi Chen, Jian Zhou, Yongjun Wang, Hao Wang, Yilong Wang

**Affiliations:** ^1^Department of Neurology, Beijing Tiantan Hospital, Capital Medical University, Beijing, China; ^2^China National Clinical Research Center for Neurological Diseases, Beijing, China; ^3^Laboratory for Oral and General Health Integration and Translation, Beijing, China; ^4^School of Stomatology, Capital Medical University, Beijing, China; ^5^Department of Stomatology, Beijing Tiantan Hospital, Capital Medical University, Beijing, China

**Keywords:** nitrate, nitrite, nitric oxide synthase, nitric oxide, ischemic cerebrovascular disease

## Abstract

Inorganic and organic nitrates are present *in vivo* and *in vitro*. Inorganic nitrate is considered a pool of nitric oxide (NO), but it can be converted into nitrite and NO through various mechanisms. It plays an important role in the regulation of complex physiological and biochemical reactions, such as anti-inflammatory processes and the inhibition of platelet aggregation, which are closely related to the pathology and treatment of cerebrovascular disease. Ischemic cerebrovascular disease is characterized by high incidence, recurrence, and disability rates. Nitrate, nitrite, and NO were recently found to be involved in cerebrovascular disease. In this review, we describe the relationship between cerebrovascular disease and nitrate metabolism to provide a basis for further advances in laboratory and clinical medicine.

## Introduction

Organic nitric esters such as nitroglycerin have been used widely in clinical and basic studies on ischemic cardiovascular and cerebrovascular diseases (1). Inorganic nitrate can also be considered a physiological source of nitric oxide (NO) *in vivo*, following transformation, especially in the vascular system (2). Advances in the field of nitrate research have shown that the physiological function of nitrate intake is not only to increase the production of NO, but also that of nitrate itself, nitrite, and other products (3). The intake or metabolism of nitrate, nitrite, and NO can affect vasodilatory and contractile functions, as well as the occurrence and development of inflammation, which is closely related to the pathogenesis of ischemic stroke and cerebral small vessel disease (CSVD).

Ischemic cerebrovascular disease, with their high disability, fatality, and recurrence rates, are a major burden on society and are a major cause of death and disability worldwide (4–6). Stroke, of which the ischemic variety accounts for the majority of events, has been ranked among the top 10 causes of global disease burden and has risen from fifth place in 1990 to third place in 2019 (7). Studies have shown that nitrate, nitrite, and NO in metabolic pathways are closely related to vascular risk factors and the mechanisms of inflammation and endothelial injury (8, 9). In this review, we discuss the involvement of nitrate metabolic pathways in different ischemic cerebrovascular disease to provide a basis for translational research for clinical application.

## Nitrate Metabolism

### Nitric Oxide Synthase

Nitric oxide was discovered as a second messenger *in vivo* by Robert F Furchgott, Murad, and Louis J Ignarro, who won the Nobel Prize. It is formed by the reaction of L-arginine with oxygen by endothelial cells, and it mediates vasodilation of vascular smooth muscle through the activation of cyclic guanosine monophosphate (cGMP), which is involved in many physiological processes. In the brain, many subtypes of its synthetic enzyme, NOS, are widely found in neurons, astrocytes, perivascular cells, and brain endothelial cells. NO and NOS regulate neurovascular coupling (NVC), which not only involves morphological connections but also dynamic functional interactions between active neurons and energy demand and supply in the blood vessels. Endothelial NOS (eNOS), calcium-independent inducible NOS (iNOS), calcium-dependent and constitutively expressed neuronal NOS (nNOS) and red blood cell (RBC) NOS are four types of NOS involved in NVC (10). The intestinal pathogen *Vibrio parahaemolyticus* converts nitrate from NO produced by host iNOS into nitrite, which acts as a signaling molecule to regulate the expression of inflammatory factors (11). The hyperactivation of nNOS after stroke can cause neurological damage, and accordingly, inhibitors of nNOS are being developed as neuroprotective agents (12, 13).

Nitrite can also regulate the production of NO by NOS, inducing vasodilation of oxygen-deficient vessels, cell protection after ischemia-reperfusion (I/R), and gene expression (14). This process is more likely to occur under conditions of ischemia and hypoxia. It can be converted into NO by xanthine oxidoreductase, mitochondrial cytochrome, deoxyhemoglobin, and myoglobin (15). Nitrite can also regulate the production of NO by NOS, thereby inducing hypoxic vasodilation, cell protection after I/R, and modulation of protein and gene expression. Some studies have shown that under severe hypoxia, nitrite, a substrate of eNOS, can generate NO in *in vitro* experiments.

### Nitrate-Nitrite-NO Metabolism

**Figure 1:**
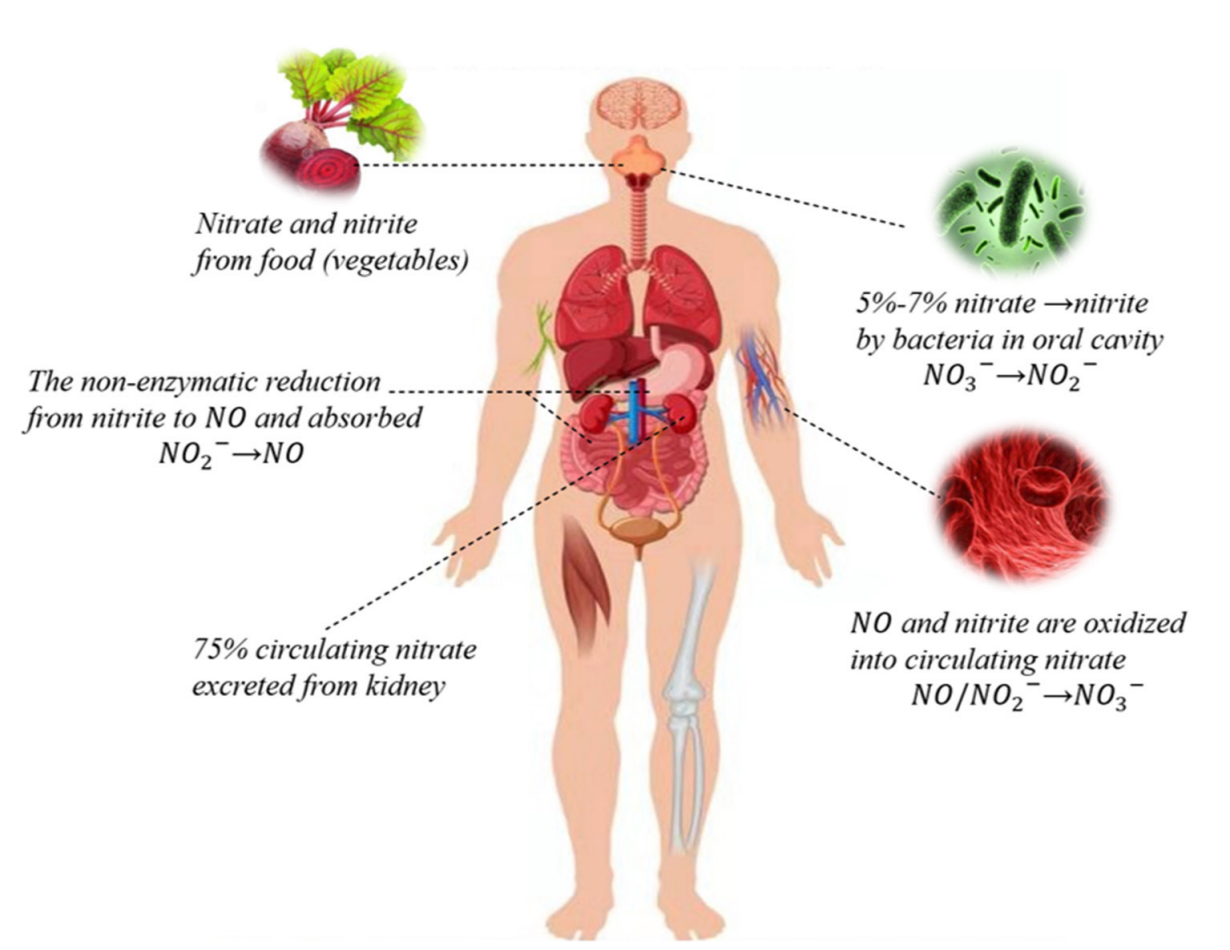
Nitrate-nitrite-NO pathways in humans. Nitrate circulating in the body is mainly derived from the dietary intake of green leafy vegetables, while a small portion of endogenous nitrate is mostly derived from oxidation reactions. Dietary nitrate is converted into nitrite by symbiotic facultative anaerobes in the oral cavity. Nitrite can be reduced to NO and both can be oxidized to nitrate. About 75% of circulating nitrate is excreted through urine, and the rest is reabsorbed through the kidneys and bile ducts or absorbed by the salivary gland that is secreted into saliva through sialin or other nonspecific transport channels.

The source of inorganic nitrate in humans is mainly food, especially vegetables and water (2). The vegetable-rich human diet provides more nitrate than the NOS enzymes produced in a day (16, 17). The nitrate from food is reduced to nitrite and then absorbed by the stomach, intestine, and other organs. Endogenous nitrites include oxidized NO and other nutrients. When bioavailability is 100%, nitrate is mainly absorbed in the proximal small intestine, resulting in a significant increase in plasma nitrate concentration within 5–6 h of intake. About 75% of the circulating nitrate is excreted through urine, and 25% is actively absorbed by salivary glands and secreted into saliva (18, 19) (Figure 1).

### Nitrate Transporters and Channels

Sialin is a polyanion transporter that mediates the H^+^-dependent transport of sialic acid, aspartate, and glutamate. Sialin has several other important roles, including its function as a nitrate transporter. Sialin is mainly expressed in salivary glands, followed by the liver and brain (20). Therefore, the transport of sialin into cells is the key first step in the nitrate metabolic pathway and plays a role in maintaining physiological functions. This step in the salivary gland also provides an unconventional pathway for the subsequent production of nitrite and NO. In addition, various transporters, such as aquaporin 6 (AQP 6) in the kidney, have been shown to function as nitrate channels in mammals (21). The permeability of chloride channels such as cystic fibrosis transmembrane conductance regulator (CFTR) and CLC-5 for nitrate is greater than that of chloride channels; however, the molecular mechanisms are not very clear (22, 23). The transport of nitrate through salivary glands to the other organs is an unconventional mode of nitrite and NO movement, especially under conditions of hypoxia and acidosis.

### Biochemical Reactions in Nitrate Metabolism

The nitrate-nitrite-NO pathway is a recently proposed complementary pathway that may be of great importance in maintaining physiological functions and the production of NO by eNOS under pathophysiological conditions (24). In normal whole blood, in the presence of oxyhemoglobin, nitrite is rapidly oxidized to nitrate, and consequently, plasma nitrate is usually much higher than nitrite. In the oral cavity, symbiotic facultative anaerobes effectively reduce nitrate in saliva from exogenous sources to nitrite through the action of nitrate reductase (2, 25). Some of these nitrites may in turn produce nitrates and NO in the digestive system. The nitrate**/**nitrite metabolic reactions *in vivo* are summarized below (26–29):

Nitrite reduction

Bacterial reduction of nitrate

$$\begin{array}{l} {{\text{NO}}_{3}}^{-}+{\text{e}}^{-}+\;{\text{H}}^{+}\to {{\text{NO}}_{2}}^{-}+{\text{H}}_{2}\text{O} \end{array}$$

Deoxyhemoglobin/myoglobin

$$\begin{array}{l} {\text{NO}}_{2}^{-}+{\text{Fe}}^{2+}+{\text{H}}^{+}\to \text{NO}+{\text{Fe}}^{3+}+{\text{OH}}^{-} \end{array}$$

Xanthine oxidoreductase

$$\begin{array}{l} {\text{NO}}_{2}^{-}+{\text{Mo}}^{4+}+{\text{H}}^{+}\to \text{NO}+{\text{Mo}}^{5+}+{\text{OH}}^{-} \end{array}$$

Protons

$$\begin{array}{l} {\text{NO}}_{2}^{-}+{\text{H}}^{+}\to {\text{HNO}}_{2}\\ {2\text{HNO}}_{2}\to {\text{N}}_{2}{\text{O}}_{3}+{\text{H}}_{2}\text{O}\\ {\text{HNO}}_{2}\;↔\;{\text{H}}^{+}+\;{\text{NO}}_{2}^{-}\\ {\text{N}}_{2}{\text{O}}_{3}↔\text{NO}+{\text{NO}}_{2} \end{array}$$

Nitrite formation

Auto-oxidation of NO

$$\begin{array}{l} 4\text{NO}+{\text{O}}_{2}+\;{2\text{H}}_{2}\text{O}\to {4\text{NO}}_{2}^{-}\;+4{\text{H}}^{+} \end{array}$$

Nitrite oxidation

Hemoglobin

$$\begin{array}{l} {4\text{NO}}_{2}^{-}+{4\text{HbO}}_{2}+{4\text{H}}^{+}\to {4\text{NO}}_{3}^{-}+4\text{Met}-\text{Hb}+{2\text{H}}_{2}\text{O}+\;{\text{O}}_{2} \end{array}$$

## Role of Nitrate Metabolism in Ischemic Stroke Risk

Common risk factors for stroke include hypertension, dyslipidemia, diabetes, smoking, alcohol, and low fruit and vegetable diet (30, 31). The pathways related to nitrate and nitrite metabolism have been shown to be involved in many risk factors in animals and humans.

Some animal experiments have demonstrated that nitrate and nitrite can affect glucose and insulin homeostasis by regulating microvascular blood flow, anti-inflammatory processes, and oxidative stress, especially when endothelial injury and eNOS dysfunction occur (32, 33). Inorganic dietary nitrate can also improve the intestinal microbiota and reduce obesity induced by a high-fat diet *via* its metabolite NO, and ameliorate glucose and lipid metabolic dysfunction in animals (34). Adding nitrate or nitrite to drugs currently used in the treatment of diabetes (such as metformin) and antiobesity drugs can also enhance their efficacy. It has been reported that mice fed a low-nitrate diet exhibit abnormality of visceral fat, dyslipidemia, insulin resistance, and perturbed endothelium-dependent diastolic function compared with mice fed a conventional diet (35). Therefore, improving the physiological function of NO by affecting nitrate-nitrite-NO homeostasis through food intake might reduce risk factors for cerebrovascular disease.

A reduction or an impairment in the utilization of NO in patients with hypertension affects endothelial function and vasodilation (36). Nitrate and nitrite, as indirect NO sources, have been the focus of an increasing number of studies on blood pressure (24). The downstream effects of nitrate and nitrite include increasing the production of NO and the modulation of signaling pathways (e.g., cGMP pathways and the activation of K^+^ channels) to promote vasodilation by endothelial cells to significantly reduce blood pressure *via* smooth muscle relaxation (37). Animal experiments have shown that nitrate can reduce blood pressure in rats with metabolic syndrome or high salt, especially in the preventive application (38–40). However, the underlying mechanisms are unclear because numerous pathways likely mediate the hypotensive effects of nitrate. The antihypertensive action of oral nitrite may involve NO and S-nitroso compounds produced in the stomach. S-nitroso compounds formed by nitrite protonation can result in the production of nitrosothiols and nitrosamines. Nitrosothiols can react with key protein mercaptans to cause protein S-nitrosylation that can strongly impact protein activity. Evidence supporting the antihypertensive action of nitrate and nitrite includes abrogation of their hypotensive effect by proton pump inhibitors (36).

Several clinical trials have shown that nitrate can affect blood pressure. A systematic review of randomized clinical trials of beetroot juice and blood pressure showed that the decrease in blood pressure after taking beetroot juice may help to reduce mortality from cerebrovascular disease, possibly *via* the nitrate-nitrite-NO pathway. However, the impact of related factors, including baseline blood pressure, overweight/obesity status, gender, and age, is unknown and warrants further investigation. Exogenous nitrate supplementation may regulate blood pressure *via* numerous mechanisms, and factors that could impact study outcome include gender, age, race, and baseline physical condition. Supplementation should be continued for at least 2 weeks to obtain sustained efficacy (37, 41). A meta-analysis confirmed these previous findings, suggesting that beetroot-derived nitrates exert antihypertensive effects, and are a safe and cost-effective nutritional supplement with potential in the management of hypertension and its complications (42). However, in a randomized controlled trial based on more than 200 samples, no blood pressure lowering effect was observed after 5 weeks of dietary inorganic nitrate supplementation. The inconsistency may be related to differences in the source of nitrate in the studies. Nitrate and other compounds in beetroot juice may have an additive effect, or, on the contrary, some compounds in green leafy vegetables may weaken nitrate's action (43). Currently, the evidence from clinical studies is not sufficient, and the conclusions need to be verified.

## Nitrate Metabolism and Ischemic Stroke

**Figure 2:**
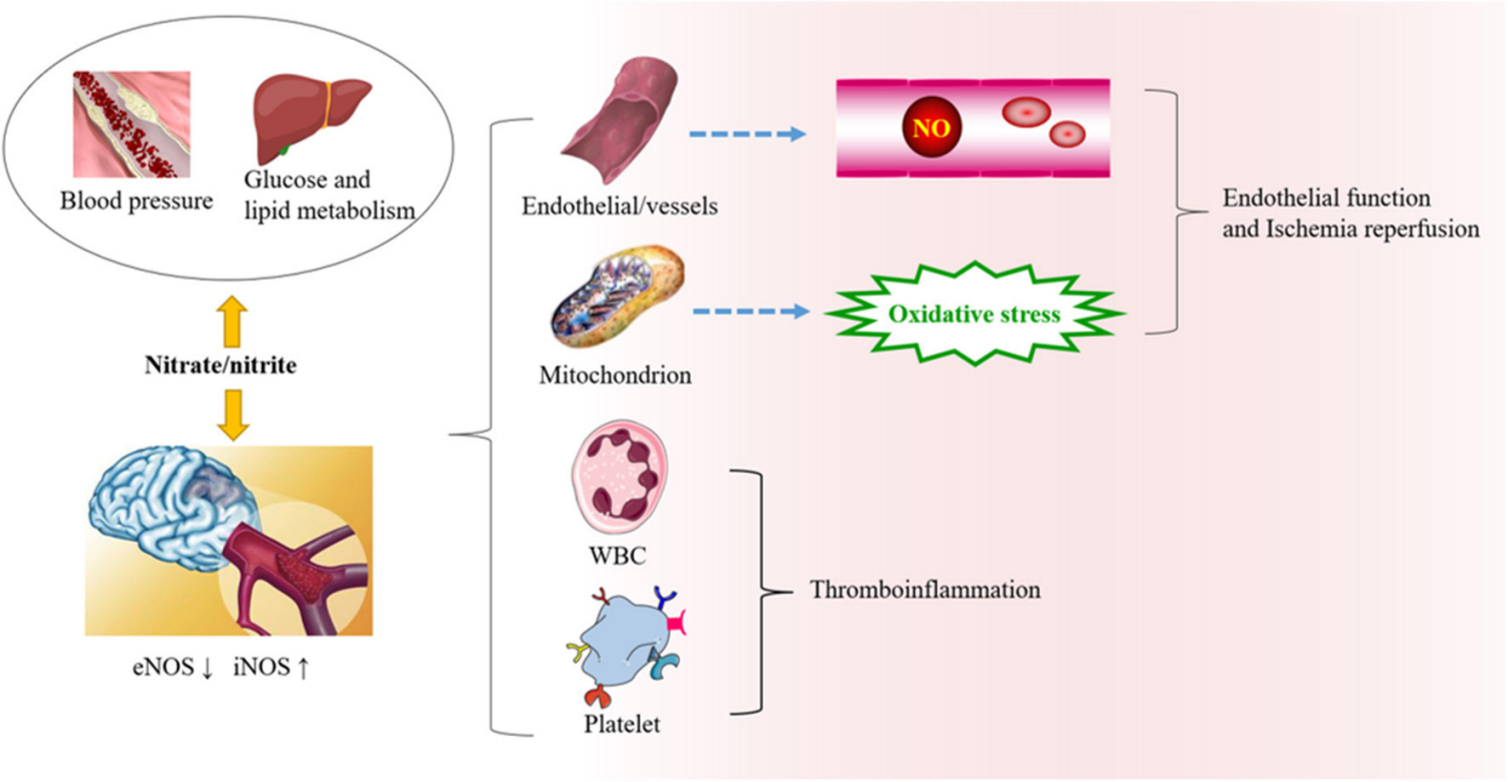
The role of nitrate/nitrite in ischemic cerebrovascular disease. In ischemic cerebrovascular disease, endothelial nitric oxide synthase (eNOS) is downregulated, while iNOS is upregulated. Beneficial NO is thereby reduced, and oxidative stress is increased. Exogenous supplementation of nitrate from food sources has great potential in protecting against oxidative damage, endothelial dysfunction, and thromboinflammation. It can also affect the risk factors related to ischemic cerebrovascular disease, such as blood pressure, blood lipid metabolism, and so on.

Ischemic stroke is closely related to the dysfunction of the vascular endothelium, platelet aggregation, and immune inflammation, and microbiotic perturbation. When the pH in tissue drops sharply because of pathophysiological processes, nonenzymatic reactions *in vivo* reduce the production of nitrite (27). During tissue hypoxia, the NO production pathway mediated by eNOS is partially inhibited (44). Compared with the traditional L-arginine pathway under aerobic conditions, the NO produced by nitrite in the reduction process is increased 6-fold (45). In addition, nitrite can also promote the synthesis and release of ATP in erythrocytes under hypoxia and stimulate eNOS to produce NO (46). The pathways involved in nitrate metabolism and stroke are summarized as follows (Figure 2).

### Endothelial Dysfunction

Research on nitrate and endothelium is mostly in the cardiovascular field but is relevant to ischemic cerebrovascular disease. Endothelial dysfunction is a core pathogenetic mechanism in ischemic cerebrovascular disease. During endothelial dysfunction, perturbation of NO synthesis or superoxide-free radical oxidation of NO increases the production of toxic substances and leads to increased vascular tension, inflammation, and adhesion of platelets and monocytes to endothelial cells, resulting in atherosclerosis (47). Thus, nitrate/nitrite increases the production of NO when NOS is compromised and exerts antioxidant stress in mitochondria.

Nitric oxide released after ischemia contributes to angiogenesis and vascular remodeling. For example, NO can induce myosin light chain dephosphorylation and increase large conductance calcium-activated potassium channel activation through the cGMP pathway and directly mediate vasodilation (48). NO can also affect the synthesis of prostaglandins and other indirect regulators. The formation of cerebral blood vessels is regulated by adjusting or forming new blood vessels (small artery anastomosis and capillaries) and remodeling small vein blood vessels, thereby enhancing the long-term prognosis of neurological function after stroke (49). Hariharan et al. found that NO in microvascular tissues was reduced in eNOS^−/−^ and eNOS^+/−^ mice, as was nitrate. In contrast, COX-2 and thromboxane A2 (TXA 2), the vasoconstrictor and platelet aggregation inducer, were upregulated in eNOS^+/−^ mice (50).

Nitrite has been applied for the treatment of I/R injury, and its target is the mitochondria in the heart and liver, where pathophysiological processes occur. In the mitochondrial respiratory chain, complex I is the most sensitive to I/R, and is, therefore, a potential target for neuroprotection in stroke. Nitrite inhibits reactive oxygen species (ROSs) overproduction through S-nitrosylation of mitochondrial respiratory chain complex I enzymes after reperfusion (51, 52). Experiments in a rat model of I/R injury showed that nitrite may exert neuroprotection by reducing ROSs production and lipid peroxidation (53). Sodium nitrite may also affect NO production and S-nitrosation and reduce neurological damage after global cerebral ischemia in mice (53, 54). However, the pathway through which nitrite mediates NO production during hypoxia remains to be determined but may involve deoxyhemoglobin, deoxymyoglobin, xanthine oxidoreductase, and acid reduction (55). In addition, ischemia increases the release of Ca^2+^ from the endoplasmic reticulum to the cytoplasm and induces endoplasmic reticulum stress and neuroinflammation. Low-dose sodium nitrite can reduce endoplasmic reticulum stress and decrease the production of ROS, thereby providing tissue protection (56).

*In vitro*, an NO donor significantly inhibits the activity of nicotinamide adenine dinucleotide phosphate (NADPH) oxidase by inducing heme oxygenase-1. Nitrate and nitrite supplementation may stimulate the NO signaling pathway, and reduce oxidative stress induced by vascular NADPH oxidase and improve endothelial function. These findings have significance for the nutrition-based prevention and treatment of vascular endothelial dysfunction (57). Current NADPH oxidase inhibitors have various limitations, including low specificity and substantial side effects. If nitrate and nitrite can inhibit NADPH in stroke treatment, it would be a simple, cheap, and relatively safe approach (58). This evidence indicates that inhibition of oxidative stress-related targets by enhancing the nitrate-nitrite-NO pathway can reduce inflammation, and may, therefore, have therapeutic potential for endothelial protection (59–62).

Oxidative stress can perturb the endothelium-dependent NO signaling pathway and impair cerebrovascular function. iNOS is also involved in the induction of inflammation after stroke. The iNOS promoter is activated after stroke, and the degree of upregulation is related to the size of the lesion (63). NO from iNOS and superoxide radicals can react to produce peroxynitrite, which can oxidize proteins, lipids, and DNA to cause extensive cell damage (64). The function of NO or nitrate in ischemic stroke is complex; whether NO is neuroprotective or neurotoxic depends on its source and redox products (65). The role of nitrate may also depend on its concentration, cation type, and exposure time (66). In conclusion, endothelial injury is the core and key step in ischemic cerebrovascular disease. Further, animal experiments and clinical trials are needed to evaluate prevention and treatment strategies (45).

### Inflammation and Thrombosis

Thrombosis is a major result of inflammation and platelet adhesion, activation, and aggregation after endothelial damage. Some small-sample studies have suggested that nitrate can inhibit platelet aggregation in healthy people and patients (67) (Table 1). For example, clinical studies have shown that a proper nitrate diet supplement (from beetroot juice) inhibits platelet aggregation in healthy volunteers. In healthy volunteers, a single supplement of 2 mmol**/**L (10.1 mg) of potassium nitrate has been shown to inhibit platelet aggregation (70). A higher nitrate intake through vegetables was related to a decrease in maximum common carotid intima thickness in a cohort study of 1,226 elderly women but was not a predictor of plaque severity. For every 29 mg/d increase in nitrate intake from vegetables, the risk of ischemic cerebrovascular disease events decreased by 17% over 14.5 years (71). In another study, dietary nitrate increased cGMP in platelets in men and improved the responsiveness to CO_2_ in cerebral vessels of men compared with women, based on a single blind, placebo-controlled crossover trial of 17 patients (74), demonstrating gender-related differences in treatment effects. There are significant differences in oral nitrate metabolism-related microbiota among individuals, and thus, gender differences in microbiota should be examined as an underlying cause (75, 76). Nitrate supplementation can also reduce platelet-derived extracellular vesicles and improve the responsiveness of patients to clopidogrel. The effect of adjuvant therapy can also be explored in patients with ischemic cerebrovascular disease because a considerable number of patients with ischemic cerebrovascular disease take clopidogrel (68). In ischemic stroke, extracellular vesicles can promote thrombosis, and an increase in these vesicles is related to the activation of platelets, which is associated with increased risk in young people (77).

**Table 1:** The effect of nitrate/nitrite on platelet aggregation in humans.

**Research objects**	**Treatments**	**Effects**	**References**
CAD patients with (*n* = 10) or without clopidogrel therapy (*n* = 10)	Received a dietary nitrate supplement or placebo	Nitrate reduced platelet-derived extracellular vesicles in CAD patients on clopidogrel therapy, increasing responsiveness to clopidogrel.	(68)
Untreated hypercholesterolemics (*n* = 69)	(250 mL) once-daily intake of dietary nitrate (beetroot juice) or placebo nitrate-depleted beetroot juice for 6 weeks	Dietary nitrate ingestion improved platelet and vascular function, associated with alterations in the oral microbiome.	(69)
Part of the study: healthy volunteers (*n* = 12)	Oral KNO_3_ (2 mmol) or KCl	Oral KNO_3_ inhibited platelet aggregation.	(70)
Elderly women (*n* = 1,226)	<53 mg/d vegetable nitrate 53–76 mg/d vegetable nitrate >76 mg/d vegetable nitrate, followed up for 14.5 years	More nitrate in vegetables can prevent carotid intima-media thickening and stroke.	(71)
Healthy older adults (*n* = 12)	140 mL of beetroot juice (containing 12.9 mmol nitrate) or nitrate-depleted beetroot juice	Nitrate supplement reduced the number of platelet aggregation in monocytes, indicating that platelet activation was decreased.	(72)
Healthy people (*n* = 24)	Nitrate of 250 mL beetroot juice or potassium nitrate capsules (KNO_3_, 8 mmol)	The intake of inorganic nitrate reduced platelet reactivity moderately in healthy men, but not in women.	(73)

*CAD, coronary artery disease*.

Leukocytes infiltrate and promote the destruction of the blood-brain barrier and cause brain parenchymal damage, which is an important contributor to cerebrovascular thrombosis and inflammation after cerebral ischemia (78). Neutrophils are the first cells to migrate from peripheral blood vessels to the ischemic area. In ischemic cerebral vessels, these cells can aggravate the ischemic injury by releasing inflammatory mediators (79). For example, cyclophilin D-mediated platelet necrosis and the ensuing neutrophil recruitment play exacerbate I/R injury after stroke (80). Neutrophil-derived NO is involved in the production of a large number of free radicals after hypoxia reperfusion (81, 82). Nitrate and nitrite not only cause vasodilation but also affect the function of leukocytes, and may, therefore, have potential in the study of thromboinflammation. Nitrite in drinking water can inhibit the adhesion and migration of leukocytes in mice fed a high-cholesterol diet and reduce inflammatory activation or stabilize atherosclerotic plaques (61). In addition to endothelial cells, iNOS in neutrophils and microglia also mediate tissue damage and further promotes inflammation, which may thus be another potential target of nitrate therapy for thromboinflammation in acute ischemic stroke (83). It is also necessary to study the effects of nitrate and nitrite on anti-inflammatory processes and endothelial protection. Reducing oxidative stress might decrease the damage by nitrate and nitrite and their products, and thereby help restore nitrate/NO metabolic homeostasis (84).

### Other Mechanisms

A recent randomized controlled trial showed that nitrate supplementation for 7 days in patients with a transient ischemic episode can reduce blood pressure and cerebral blood flow, and improve cerebral autoregulation, without affecting cerebrovascular CO_2_ responsiveness (85).

Microbiota is a research hotspot in cerebrovascular disease, especially ischemic cerebrovascular disease. Microbiota may also be related to nitrate metabolism. Nitrate from food, in addition to being transformed into nitrite by microorganisms in the body, has a regulatory effect on human microbiota. Clinical studies show that consuming fruit and vegetable juice containing nitrate for 3 days has a significant effect on the two most abundant phylum of bacteria, *Firmicutes* and *Bacteroidetes*, in feces (86). A high ratio of *Firmicutes, Bacteroidetes* is considered as a sign of intestinal disorder. After an experimental stroke in young mice, the microbial community changes, similar to that in uninjured old mice, in which the ratio of *Firmicutes, Bacteroidetes* is 9-fold higher than in the young mice (87). *NOS2* (iNOS gene) and oxidative stress-related genes are upregulated in acute ischemic stroke. iNOS-derived NO reacts with oxides actively produced by oxidative stress genes to generate nitrate, which destroys the intestinal barrier and causes *Escherichia coli* to play a proinflammatory role in the anaerobic intestinal environment, thereby aggravating inflammation (88).

## Nitrate Metabolism and Cerebral Small Vascular Disease

The cerebral small vascular disease mainly affects the cerebral small arteries, small veins, and capillaries (8). CT or MRI shows lacunae, short-term subcortical small infarcts, high intensity of the white matter, enlargement of the perivascular space, microbleeds, and brain atrophy (89, 90). At present, the pathophysiological mechanisms of CSVD are not clear but are the focus of the current research. CSVD may be related to chronic ischemia/hypoperfusion, blood-brain barrier damage, and endothelial dysfunction (91–93). CSVD is a relatively unrecognized chronic disease, but its burden is very high, causing between a fifth and a quarter of strokes and nearly half of cognitive impairments (94).

### Sporadic Cerebral Small Vessel Disease

A cross-sectional study showed that arterial stiffness is related to imaging markers of small vessel diseases, including high white matter signal, and cerebral microhemorrhage (95). Arterial stiffness is a pathological factor in vascular diseases, especially of small vessels and microvessels. The most fundamental pathological feature in cerebrovascular disease, especially in CSVD, is cerebral atherosclerosis, similar to that in large arteries. NO impacts arterial stiffness by affecting smooth muscle relaxation and vascular tension. The nitrate-nitrite-NO pathway can notably ameliorate arterial pressure in the endothelium and improve arterial stiffness. At present, it is considered that high white matter signals may be related to chronic ischemia and age. Dietary high nitrate does not change overall cerebral perfusion in older adults, but does increase local cerebral perfusion in the frontal white matter, and may play an important role in the improvement of executive and cognitive functions (96).

Inducible nitric oxide synthase, a risk factor for CSVD, can barely be detected in the normal and healthy brain, except in the elderly. Its expression in the elderly is upregulated by cytokines or inflammatory stimulation (64), which may aggravate CSVD *via* oxidative stress by NO and nitrate. Intron 4 insertion a/deletion b genotype of eNOS is associated with simple lacunar infarction. Studies have shown that protection by the 4a variant might involve modulating eNOS promoter activity and increasing NO levels. The pathological change caused by NO deficiency is local microangioma of proximal arteries rather than diffuse arteriosclerosis of distal arteries (97). The two subtypes of simple symptomatic lacunar infarction and nonischemic white matter hyperintensity may show heterogeneity. There are some studies on the functions of introns 4a/b in the eNOS gene and their association with CSVD and its risk factors, but the findings have been inconclusive. Some studies have shown that intron 4a has a protective effect on cerebral vessels, while others have suggested that its effect may be related to other phenotypic differences. Furthermore, the intronic 4a allele has been suggested to be a risk factor for lacunar infarction and white matter hyperintensity. These discrepant findings may reflect differences in race and genetic background (97). Therefore, the role of the eNOS gene in different phenotypes of ischemic cerebrovascular disease needs to be further studied and more attention has to be given to whether the sialin gene has a synergistic effect.

### Hereditary Cerebral Small Vessel Disease

In Fabry disease, the X-linked lysosomal storage CSVD disease, the pathogenetic mechanism is endothelial dysfunction (98), where the lack of α-galactosidase leads to multiple system defects (99). The level of plasma nitrate in patients with Fabry disease is lower than that in controls, although not statistically significant. The decrease in plasma nitrate may be associated with a change in NO regulation by the endothelium. The metabolic abnormality in this disease may increase NO and protein nitration in local tissues, and the excessive generation of superoxide is likely to be a contributing pathogenetic factor. The lack of α-galactosidase in Fabry disease may account for the excessive production of NO in tissues and the high ROS levels. NO reacts with ROS to form peroxynitrite, which oxidatively reacts with key enzymes and amino acids, leading to cell dysfunction and sometimes death. At the cellular level, NO competes with superoxide dismutase to scavenge ROS. The increase in NO content in tissues may lead to cellular peroxidation by excessive peroxynitrite and nitrotyrosine (100).

## Nitrate Metabolism and Cognition

Vascular dementia is a cognitive impairment caused by cerebrovascular disease (especially ischemic cerebrovascular disease), but there are no licensed treatments for the disorder. The relationship between cerebrovascular pathology and cognitive impairment in vascular dementia is controversial, and the underlying mechanisms need to be further studied (101). Studies have shown a link between nitrogen oxide levels and dementia. The levels of serum nitrogen oxides are significantly lower in patients with dementia, especially in patients with vascular dementia (102).

Nitric oxide synthase and NADPH oxidase may have inflammatory roles in vascular dementia and are important targets in the treatment of vascular dementia (103). Oxidative stress is an important pathogenetic mechanism in vascular dementia and is related to damage to the NOS pathway and endothelial dysfunction (104). Some studies have shown that vascular dementia is an aging-related disease. In one study, nitroglycerin (an NO donor) treatment significantly recovered vasodilatory function in elderly mice. After the application of L-NAME (an eNOS inhibitor), the therapeutic effect in young and old mice disappeared, indicating that endothelial dysfunction may also occur in vascular dementia (105). Therefore, nitrate as an inorganic NO donor is worthy of further study in the treatment of vascular dementia.

Anterior cerebral blood flow in patients with vascular dementia is decreased, as shown by imaging studies (106). It was reported that beetroot juice can regulate the cerebral blood flow response to task performance in healthy adults and improve cognitive ability (107). Therefore, the mechanism of vascular dementia may be related to an imbalance in nitrate metabolism, and nitrate supplementation may improve blood flow and brain function, and inhibit inflammatory processes.

## Safety of Nitrate and Nitrite

With the discovery of the nitrate-nitrite-NO pathway and its physiological significance, inorganic nitrate is now considered a natural NO precursor, and therefore, its beneficial systemic physiological actions deserve attention. The safety of nitrate and nitrite has always been controversial (108). According to the European Food Safety Agency, the average nitrate intake in adults is about 157 mg/day (109). The WHO acceptable daily intake (ADI) for nitrate is 0–3.7 mg/kg, which is equivalent to 222 mg of nitrate in an adult of 60 kg (110). The so-called carcinogen is actually nitrosamine (111). There is still insufficient evidence that nitrite and nitrate in food can cause cancer (112). Nitrate supplementation from beetroot can also increase the level of NO metabolites in circulation, increase salivary pH value, and significantly change oral flora, which is beneficial to oral health, as demonstrated by a single-blind randomized crossover study. This study has significance for the prevention and treatment of periodontitis and caries (113). In fact, with the popularity of a healthy lifestyle and the increase in daily vegetable intake, the reasonable dietary safe dose of inorganic nitrate and its beneficial physiological effects need to be evaluated by higher level animal and clinical trials. Thus, while it is considered a safe and good choice to acquire nitrate from food sources, the optimal doses and effectiveness for different diseases need to be investigated.

### Prospects

In cardiovascular and cerebrovascular diseases, endothelial injury is an important etiopathological factor, and a reduction in NO production is a key problem (2, 114). The safest and most convenient source of exogenous nitrate is food, especially vegetables. There are also prospects for research on nitrate diet patterns, such as the Mediterranean diet (115). Healthy diet management is an important means of stroke prevention and treatment. Diet habits such as high-salt and high-fat diets are risk factors for stroke. Studies have shown that adhering to the Mediterranean diet can reduce the risk of stroke, although the findings are controversial. In addition, eating more plant-based foods may reduce the risk of stroke recurrence. Vegetables account for a considerable proportion of these healthy dietary patterns, and the benefits of vegetables may include nitrate and NO (116, 117).

Drug use in humans is more complicated than in animals. Long-term infusion of large doses of nitrite is needed to effectively reduce vasospasm-related subarachnoid hemorrhage in animal studies, but the dose of nitrite required for protection in the model of I/R injury is very low (29). It is a research objective to provide nitrate and nitrite to experimental animals and evaluate their effect on I/R injury and the protection of multiple organs, and then carry out clinical trials. Small-sample preliminary findings suggest that intravenous nitrite is safe for patients with cardiac arrest. However, a randomized controlled trial involving more than 1,000 patients showed that sodium nitrite treatment did not significantly improve the final hospital survival rate of patients without hospital cardiac arrest (118). In the nitrate metabolic pathway, there is heterogeneity caused by differences in heredity, gender, and age. Therefore, personalizing drugs and their doses for all patient types still requires further study.

The nitrate cycle is affected by many factors. Homeostasis may be affected by dietary habits. If the intake of multiple types of foods and drinks containing polyphenols, which are abundant in red wine, apples, and black tea (119), is increased, a reduction in nitrite can occur in the stomach, thereby reducing blood pressure. However, the effect can be weakened by drugs such as proton pump inhibitors (24). Studies have shown that the intake of sodium nitrite can significantly reduce systolic blood pressure and that esomeprazole pretreatment can eliminate this effect, indicating that the acidic conditions of the gastric cavity are needed for the blood pressure lowering action. The effect of esomeprazole may be related to a reduction in acidity and a decrease in hydrogen ions, which likely affects the nitrate-nitrite-NO pathway (120, 121). Being healthy has become a popular concept, particularly the concept of oral health. However, the antibacterial mouthwashes that are commonly used are not necessarily beneficial to whole-body health. While oral antibacterial solutions can kill bacteria in the mouth, they can also decrease the conversion of nitrate to nitrite, and thereby reduce the beneficial effect of exogenous nitrate intake on general health, including the antihypertensive effect (2, 120). Therefore, nitrate-reducing bacteria play a significant role in general health by modulating the nitrate-nitrite-NO pathway, and the mechanisms of protection and disease may be a major target of future research (122).

Further studies are required to clarify the link between nitrate/nitrite and cerebrovascular disease. The etiopathogenesis of ischemic stroke and CSVD are very different, but the risk factors are similar to some extent, and both involve microcirculatory disorder, endothelial damage, platelet aggregation, and inflammation. Future studies on nitrate/nitrite and ischemic cerebrovascular disease should identify specific therapeutic targets. Nitrate, nitrite, NO, and their derivatives may play different roles in different stages, and those derived from different sources may influence different pathways. Furthermore, tracing the metabolism of exogenous nitrate supplements and conducting high-level clinical trials should help test and validate current concepts. For the prevention of vascular diseases and their complications, the effects of long-term nitrate supplements need to be investigated by large-scale clinical trials. Currently, there is no safety concern in the intake of these inorganic substances from food at a reasonable dose. Finally, although the nitrate-nitrite-NO pathway is now considered to participate in extremely important physiological processes, research on the nitrate transport-related gene *SLC17A5* is still lacking. In addition, while sialin is highly expressed in the brain, it is only known that it primarily transports sialic acid and nitrate and that its loss of function can cause heritable disease, even death. Therefore, it has broad research prospects, such as population genetics and the expression of the *SLC17A5* gene, whether different types of ischemic cerebrovascular disease are associated with *SLC17A5* and the relationship between sialin and NOS.

## Conclusion

There is convincing preliminary evidence for the application of nitrate in both animal experiments and human intervention. Nitrate is particularly important to human physiological and pathophysiological states, and there are potential targets in the vascular system (45). Genes involved in nitrate/nitrite metabolism are related to cerebrovascular disease, and chemical compounds could play an important therapeutic role by providing NO. Nitrate supplementation may be a convenient, economic, and effective way to reduce the risk of ischemic cerebrovascular disease. Furthermore, adjuvant treatment with exogenous supplements or increasing the transformation of nitrate in pathophysiological states may inhibit inflammation and protect the endothelium.

## Author Contributions

All authors listed have made a substantial, direct, and intellectual contribution to the work and approved it for publication.

## Funding

This research was supported by grants from the National Natural Science Foundation of China (81825007, 81901177, and 81971091), Beijing Outstanding Young Scientist Program (No. BJJWZYJH01201910025030), National Key R & D Program of China (Nos. 2017YFC1307900 and 2017YFC1307905), Beijing Hospitals Authority Youth Programme (QML20200501 and QML20190501), Beijing Municipal Science and Technology Commission (Nos. D17110700300000 and D17110000301700), Beijing Tiantan Hospital, Capital Medical University (2018-YQN-1 and 2020MP01), Beijing Excellent Talents Training and Supporting- Top Youth Team by Beijing Municipal Science and Technology Commission (No. 2016000021223TD03), Youth Beijing Scholar Program (No. 010), Beijing Talent Project - Class A: Innovation and Development (No. 2018A12), and Young Elite Scientist Sponsorship Program (2019QNRC001).

## Conflict of Interest

The authors declare that the research was conducted in the absence of any commercial or financial relationships that could be construed as a potential conflict of interest. The reviewer YW declared a shared affiliation with the authors to the handling editor at time of review.

## Publisher's Note

All claims expressed in this article are solely those of the authors and do not necessarily represent those of their affiliated organizations, or those of the publisher, the editors and the reviewers. Any product that may be evaluated in this article, or claim that may be made by its manufacturer, is not guaranteed or endorsed by the publisher.
